# Safe surgical technique: cement-augmented pedicle screw instrumentation and balloon-guided kyphoplasty for a lumbar burst fracture in a 97-year-old patient

**DOI:** 10.1186/1754-9493-7-3

**Published:** 2013-01-08

**Authors:** Thomas Freude, Benjamin König, Frank Martetschläger, Sebastian Siebenlist, Markus Neumaier, Ulrich Stöckle, Stefan Döbele

**Affiliations:** 1 Department of Traumatology, Eberhard Karls Universität Tübingen, Schnarrenbergstrasse 95, Tübingen, 72076, Germany; 2 Department of Traumatology, Klinikum Rechts der Isar, Technische Universität Muenchen, Ismaninger Strae 22, Munich, 80809, Germany

**Keywords:** Thoracolumbar fracture, Augmented screws, Kyphoplasy, Spine fracture in elderly

## Abstract

**Background:**

During the last few years, an increasing number of unstable thoracolumbar fractures, especially in elderly patients, has been treated by dorsal instrumentation combined with a balloon kyphoplasty. This combination provides additional stabilization to the anterior spinal column without any need for a second ventral approach.

**Case presentation:**

We report the case of a 97-year-old male patient with a lumbar burst fracture (type A3-1.1 according to the AO Classification) who presented prolonged neurological deficits of the lower limbs - grade C according to the modified Frankel/ASIA score. After a posterior realignment of the fractured vertebra with an internal screw fixation and after an augmentation with non-absorbable cement in combination with a balloon kyphoplasty, the patient regained his mobility without any neurological restrictions.

**Conclusion:**

Especially in older patients, the presented technique of PMMA-augmented pedicle screw instrumentation combined with balloon-assisted kyphoplasty could be an option to address unstable vertebral fractures in “a minor-invasive way”. The standard procedure of a two-step dorsoventral approach could be reduced to a one-step procedure.

## Background

Burst fractures of the thoracolumbar spine have the highest incidence rate of spine fractures. There is a consensus that stable fractures are treated conservatively [1]. For unstable burst fractures, the surgical approach still presents a topic of discussion [2-4]. The posterior spinal instrumentation with pedicle screws allows an indirect fracture reduction and prevents consecutive kyphosis [5,6]. Due to a high prevalence of early instrumentation failure, an additional stabilization of the anterior spinal column with strut grafting, plates, and mesh cages via a second anterior approach has been reported with sufficiently positive clinical results [4,7-9]. The second approach, however, is associated with an increased morbidity, a longer operation time, higher blood loss and high donor site morbidity [2,10].

In the recent literature, a new technique, in which dorsal instrumentation is combined with a balloon kyphoplasty in order to address these problems, has been introduced. For acute traumatic compression or burst fractures, promising clinical results have been demonstrated in young adults when absorbable bone cement was used for the kyphoplasty [4,11-13]. But especially in older patients with an osteoporotic bone structure, an integration of absorbable bone cement cannot be expected [14].

This case report describes the safe technique in 97-year old patient with a lumbar burst fracture and neurological deficits, treated by cement-augmented pedicle screw instrumentation and balloon-guided kyphoplasty. To our knowledge, this surgical procedure has not yet been reported in a patient of such a high age and accompanying neurological symptoms.

## Case presentation

**Figure 1:**
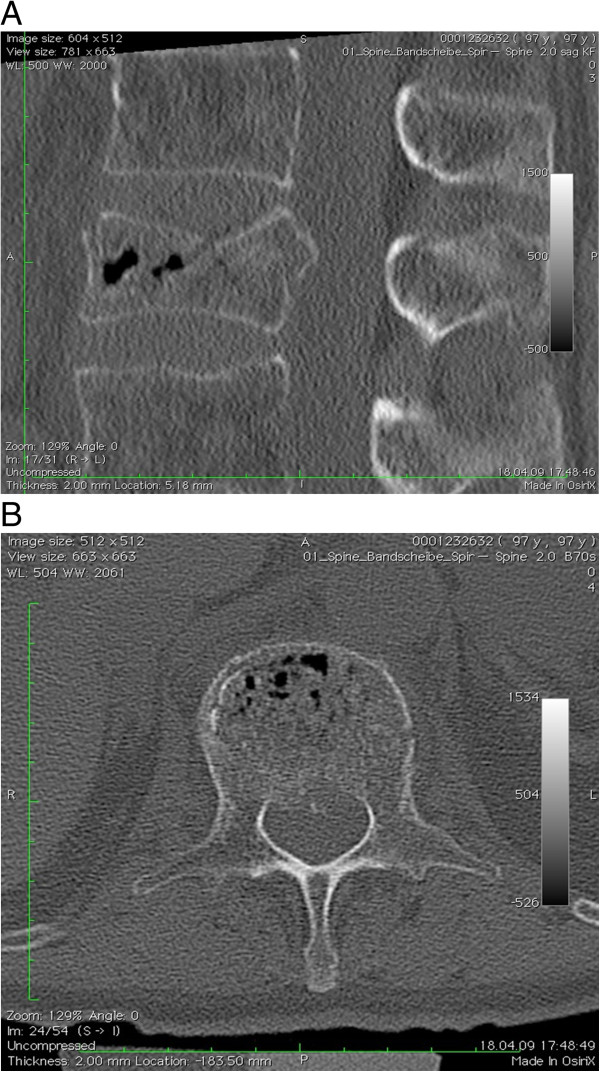
**A to 2-C CT scan of the spine of the 97-year-old patient who presented with an incomplete motoric paraplegia (Frankel grade C) associated with an L 2 burst fracture. A** Preoperative sagittal reconstruction. **B** Preoperative axial CT scan showing 50% canal compromise. The patient was managed with near-anatomic reduction without any direct reduction of the fracture fragment.

A 97-year-old man in good age-corresponding condition had fallen from a standing height. At the time of his initial visit at our hospital, the CT scans indicated a multifragmentary endplate fracture involving the posterior edge of the L2 vertebral body as well as a considerable narrowing of the spinal channel (Magerl Classification type A3-3.1 [3]). Initially, the patient denied surgical intervention. At this time, no neurological deficit was detected. Because of severe pain and a progressive weakness of the lower limbs - grade C according to the modified Frankel/ASIA score [15] - the patient attended our department again on the following day. The follow-up CT scan revealed a progression of the vertebral compression and an increased bulging of the dorsal wall into the spinal cord space (Figure 1).

Because of the patient’s good age-corresponding condition, it was decided to perform a posterior decompression by distraction and lordosation using an augmented pedicle screw instrumentation with non-absorbable bone cement. In a one-step procedure, an additional balloon-assisted kyphoplasty was performed in order to support the anterior column (see below: **Safe Surgical Technique**). The operative time was 72 min. Intraoperatively, there was no complication. A 48-hour monitoring which was carried out after the surgical intervention at the intensive care unit was uneventful. The monitoring in the ICU was only due to the advanced age of the patient and was outside the normal treatment guidelines. No sensomotoric deficit was detected during the wholein-patient-stay-grade E according to the modified Frankel/ASIA score [15]. The day after the surgical procedure, the patient was started with doing exercises, including having dinner while sitting upright in his bed. During his physical therapy, the patient was fairly pain-free (VAS = 3), and moved around using a walking frame. Ten days after the surgery, the patient was discharged. Due to the poor bone quality, that had been discovered during the insertion of the pedicle screws, and the obvious senile osteoporosis, we started with an oral anti-osteoporosis therapy with the application of bisphosphonate 75 mg/week, calcium 1000 mg/d and cholecalciferol 500 mg/d, with the advice that it should be continued after the patient had been sent home.

**Figure 2:**
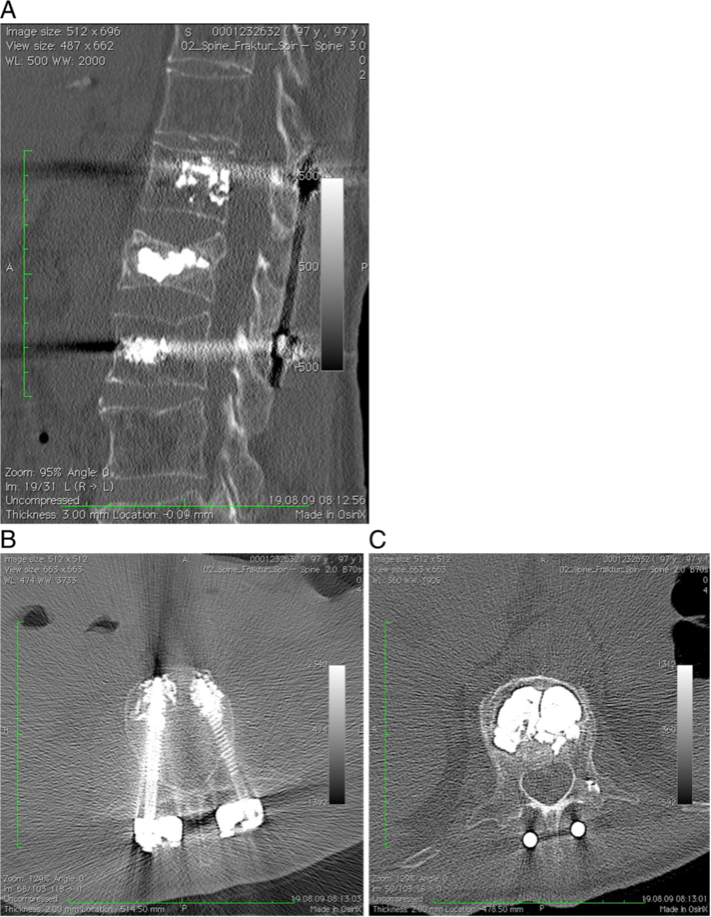
**A Sagittal reconstruction CT scan showing the well-maintained restoration of the vertebral body height r and sagittal alignment with augmented cephal and caudal screws. B** Cement augmentation of the pedicel screws. **C** Axial CT scan showing no residual canal stenosis and the filling of the vertebral body with PMMA cement through the kyphoplasty technique.

At 12 months follow-up the patient is completely remobilized and had regained his pre-injury level of activity. He is able to walk over a distance of 2 km without any pain. He is taking part in his normal social life and is living in complete independence without any need of support (Table 1). CT-scans (Figure 2) showed no secondary dislocation of the screws and no signs of instability in the kyphoplasted vertebra.

**Table 1:** **Showing the Oswestry**[16-18]

	***Pre-admission***	***Admission***	***24 h post OP***	***12d post OP***	***6 months post OP***	***12 months post OP***
Oswestry (%)	100	0	22	48	93	93
VAS	0	10	5	2	0	0
Frankle/ASIA	E	C	E	E	E	E
Morpine	-	+	+	-	-	-
Analgetics	-	+	+	+	-	-
Calcium	-	-	-	1000 mg/day	1000 mg/d	1000 mg/d
Bisphosphonates	-	-	-	75 mg/week	75 mg/week	75 mg/week
Cholecalciferol	-	-	-	500 mg/day	500 mg/day	500 mg/day

### Safe surgical technique

The surgery was performed under general anaesthesia. The patient was placed prone on a radiolucent table. A partial reduction of the kyphotic deformity was obtained under image intensifier control. For dorsal instrumentation, the Synthes USS system (Synthes, Solothurn, Switzerland) was used. Four Schanz pedicle screws (diameter of 6 mm, thread length of 45 mm) were placed transpedicularly on both sides into the body of the non-fractured vertebrae cephalad and caudad to the fractured vertebra, through the use of a standard longitudinal midline incision. Afterwards, the screws were removed and the prepared non-absorbable cement (KyphX**® HV-R™**, Kyphon Inc., Sunnyvale, USA) was applied into the non-fractured vertebra with a trokar in order to ensure a greater stability of the screws in the osteoporotic bone. After the cement had completely hardened, the screws were connected with two titanium rods with a length of 7.4 cm. Further fracture reduction was obtained through the use of distraction tools in order to restore anatomic alignment under the control of an image intensifier. The fragments may have occurred as a result of ligamentotaxis. The fracture fragments were neither exposed or directly manipulated, nor was any kind of open reduction, decompression laminectomy or laminotomy performed. After that, the kyphoplasty of the fractured first lumbar vertebra was carried out with the KyphX-balloon system (Kyphon, Sunnyvale, California, USA). At first, the pedicle finder was used in order to open both pedicles of the fractured vertebra and to create a transpedicular pathway within the fractured vertebral body. This opening of the pedicles was carried out under image intensifier guidance. Next, two trokars with a diameter of 4.2 mm were inserted in the pre-drilled pedicle pathways. The trokars were used as guidance for the two 20 mm kyphoplasty balloons. Under image intensifier monitoring, both were inflated up to 150 psi and 4 ccm. During this procedure, the dorsal wall of the vertebral body was carefully observed in order to avoid its dislocation into the spinal cord space. After the normal vertebral height had been restored with the kyphoplasty balloons, the newly-formed cavity was carefully filled with non-absorbable cement (KyphX**® HV-R™**, Kyphon INC., Sunnyvale, USA) under biplanar control. The wound cavity was flushed and haemostasis was achieved. The intraoperative blood loss amounted to 200 ml. The patient did not require any intra-/or postoperative transfusion of blood products.

## Conclusion

Thoracolumbar burst fractures still are a great challenge to surgeons, especially in elderly patients with osteoporotic bones. In old and very old patients, early mobilization has to be the primary goal in order to reduce the time of the hospital stay and to decrease the morbidity and the mortality respectively. The short-segment posterior pedicle screw instrumentation is a well-accepted technique for the reduction and stabilization of vertebral burst fractures [4,11,19]. According to the long-term follow-up results that have been gathered over the last decades, the exclusive stabilization with this technique seems to be insufficient. The reported failure rate varies from 20 to 50% in the case of pedicle screw failure and a consecutive increase of spinal kyphosis [8,20]. Therefore, additional anterior procedures are recommended. The anterior approach allows a full visualization of the fracture. The use of plates, cages or iliac cortical grafts facilitates the direct vertebral restoration, the decompression and a solid fusion. Although the anterior approach leads to an extended operation time, the more traumatic ventral approach, on the contrary, leads to a higher blood loss and an elevated postoperative morbidity.

During the last decade, the dorsal instrumentation in combination with absorbable cement augmentation techniques has become increasingly popular for younger patients [13,14,19]. Marco and Kushwaha [4] treated 39 patients with a mean age of 38 years, with or without a neurological deficit, with a short-segment instrumentation in combination with a calcium phosphate cement reconstruction. They claimed that an excellent lasting reduction of unstable burst fractures had been achieved during a 2-year-follow-up with the use of this combined technique. Nevertheless, some complications were observed: Screw breakage was recorded in two patients, wound dehiscence was observed in one patient and finally a pseudarthrosis of the dorsolateral fusion in another. Cement leakage was not observed at all though. However, patients with senile osteoporosis were excluded. Cho et al [12]. reported on a group of 70 patients (mean age 45 years) 50 of whom were treated by short-segment pedicle-screw instrumentation alone while the remaining 20 were treated by combined reinforced PMMA vertebroplasty. In the first group, implant failure was observed in 22% of the patients and after a two-year follow-up, an increase of the kyphotic deformity of 6.2 degrees was recorded. No implant failure was observed in the second group, where the increase of the kyphotic deformity was amounted to only 0.3 degrees. In 1998, Mermelstein et al [21]. already conducted a biomechanical study in which the reinforcement of the thoracolumbar burst fractures with calcium phosphate cement in a cadaver model of an L1 burst fracture was observed. A decrease of the pedicle-screw bending moments of 59% in flexion and of 38% in extension was observed. Therefore, it was concluded that this combined technique may improve the patient’s outcome without a secondary anterior procedure. In a study of Verlaan et al [13,14]. the height of the fractured vertebral body could be restored at up to 91% of the estimated intact height in 20 patients with transpedicular balloon vertebroplasty in combination with posterior instrumentation. Korovessis et al [2]. included 23 patients with a mean age of 48 years in a prospective study with balloon kyphoplasty using calcium phosphate and stabilization with pedicle screw instrumentation and fusion. After a follow-up of 24 months, an improvement of the kyphosis deformity as well as of the vertebral body weight ratio was reported.

Only a few cases of dorsal internal fixation in combination with cement augmentation techniques for older patients with osteoporotic bones, have been reported in the current literature [22-24]. The case of a 97-year-old male with a lumbar burst fracture that is presented here, illustrates the use of a dorsal pedicle screw instrumentation not only in combination with a balloon-assisted kyphoplasty but also with additional cement-augmented pedicle screws. To our knowledge, this technique has not yet been reported for a patient of this age. For this one-step-procedure with a non-absorbable bone cement augmentation, we decided to provide more stability in a severe osteoporotic bone in order to prevent secondary cutting-out or screw loosening. Our patient recovered quickly and could be remobilized without any pain. Because of the use of this technique, no additional anterior approach was necessary. Therefore, this technique lead to a reduction of the operative trauma and the risk of postoperative wound complications as well as of the morbidity and the length of the stay in hospital. Wound infection or necrosis did not occur at all. At this high age, multiple complications can result in a prolonged wound healing. Local infection rates after dorsoventral approach ranged between 7% and 14% [25-27]. The next problem consists in the osteoporotic vertebrae themselves: We were able to observe fractures of the vertebrae in the next segment that was located caudal or cephalad to the fixateur interne. These fractures were manly located cephalad, because of the increased stress and forces that were caused the stabilisation. It should always be discussed how many segments have to be addressed in order to provide enough stability to prevent the next fracture. The use of cement to augment the screws provides more stability for the osteoporotic bone, but the removal of the screws is very problematic and associated with a high incidence of collateral damage.

Especially in older patients, the presented technique of PMMA-augmented pedicle screw instrumentation combined with balloon-assisted kyphoplasty could be an option to address unstable vertebral fractures in “a minor-invasive way”. In order to minimize the surgical morbidity and to increase the quality of life, the standard procedure of a two-step dorsoventral approach has to be reduced to a one-step procedure.

## Consent

Written informed consent has been obtained from the patient for the publication of this case report and any accompanying images. A copy of the written consent is available for review for the editor-in-chief of this journal.

## Competing interests

The authors declare that they have no competing interests. There are no financial interests by any of the authors with regard to the companies whose products are described in this paper, i.e. Kyphon/Medtronic and Synthes.

## Authors’ contribution

All authors have made a significant contribution to the different steps of the processing of the patient’s history as well as to the writing and the editing of the manuscript. TF and SD have conceived the idea for the study and have written the first draft. SS has provided research support and advice throughout the project. Furthermore, FM and BK have provided expertise in artwork. The corresponding author TF and US have carried out the surgical procedures and have provided geriatric expertise. All authors have read and approved the final manuscript.
